# Retrograde Robotic Radical Prostatectomy: Description of a New Technique and Early Perioperative Outcomes

**DOI:** 10.1155/2014/945604

**Published:** 2014-03-10

**Authors:** Gino J. Vricella, Zachary Klaassen, Martha K. Terris, Rabii Madi

**Affiliations:** ^1^Urology Institute, University Hospitals Case Medical Center, Case Western Reserve University, Cleveland, OH 44106, USA; ^2^Department of Surgery, Section of Urology, Georgia Regents University, 1120 15th Street, BA 8414, Augusta, GA 30912, USA

## Abstract

*Objectives*. This research was conducted to describe a novel technique for performing robotic-assisted laparoscopic prostatectomy (RALP) using a retrograde approach that mimics the classic open surgical technique. *Methods*. From June 2009 to March 2011, we performed 18 nonconsecutive RALPs using a novel retrograde approach. Patients were initially selected with D'Amico low to intermediate risk disease. Pre-, intra-, and postoperative data were analyzed in all patients. *Results*. All 18 patients had successful surgery without any intraoperative complications. Mean preoperative PSA was 6.0 ng/mL. Nine patients had biopsy Gleason score (GS) 6, seven patients had GS 7, and two patients had GS 8. Fourteen patients had clinical stage T1c and four patients had stage T2a. Mean operative time was 198 minutes, with a mean robotic console time of 168 minutes. Fifteen patients had T2 disease on the final pathology and three had T3 disease. With a median follow-up of 11 months, 10 patients had an undetectable PSA. *Conclusions*. Our early experience with retrograde robotic-assisted laparoscopic prostatectomy demonstrates the feasibility of this approach with early outcomes comparable to the contemporary antegrade approach. Long-term study with a greater number of patients will be necessary to fully evaluate the oncologic and functional outcomes using this technique.

## 1. Introduction

Following the initial description of robotic-assisted laparoscopic prostatectomy (RALP) in 2001 [1], this procedure is now the most common surgical procedure performed in the United States for treatment of localized prostate cancer [2]. The advantages of RALP over the classical open radical retropubic prostatectomy (RRP) approach include decreased operative blood loss, decreased need of analgesics, earlier convalescence, and superior cosmesis [3, 4]. Among the various challenges facing the surgeon interested in adopting the robotic approach is the alteration in surgical technique required to perform RALP successfully. This adaptation may be of minimal significance to urology residents and fellows exposed and trained in minimally invasive techniques; however, it may be an obstacle for surgeons who have performed open RRP for years and desire learning the robotic technique.

One of the major differences between the technique currently used for RALP and the open RRP is the direction of prostate dissection. Contemporary RALP has adopted the original laparoscopic approach as described by Guillonneau and Vallancien [5]. Using this technique of antegrade dissection, the prostate is initially incised at its junction with the bladder neck anteriorly. After opening the bladder neck, the prostate is incised posteriorly to the level of the vas deferens and seminal vesicles. The vas deferens and the seminal vesicles are then controlled and the prostate is dissected off the rectum in an antegrade fashion until the apex of the prostate is reached. The prostatic pedicles are divided and a lateral incision is made in the periprostatic fascia. Nerve sparing, if considered, is usually performed during this stage of the surgery and dissection of the neurovascular bundles (NVBs) is performed from these lateral incisions.

Open RRP, as initially described by Walsh almost three decades ago, differs significantly from RALP in the direction of dissection [6]. When contemplating nerve sparing, the lateral prostatic fascia is initially incised and the neurovascular bundles are identified and dissected posteriorly. The apex is then incised and the urethra is exposed and cut. Further dissection is performed in a retrograde fashion using sharp and fine blunt dissection up to the level of vas deferens and seminal vesicles and release of the NVBs is completed. The objective of this paper is to describe a novel retrograde approach to RALP, which replicates the open RRP technique as initially described by Walsh et al. [6].

## 2. Materials and Methods

### 2.1. Patient Selection and Perioperative Variables

We performed a retrospective review of a prospectively maintained urologic oncology database. We received Institutional Review Board approval to identify 18 nonconsecutive patients who underwent RALP using the retrograde approach between June 2009 and March 2011. Patient selection was limited to those patients with smaller prostates (less than 50 grams) and low volume cancer. Preoperative variables included mean age, mean Body Mass Index (BMI), mean International Prostate Symptom Score (IPSS), mean Sexual Health Inventory for Men (SHIM), biopsy Gleason score, and clinical T stage. Operative variables included mean operative time, mean robotic console time, nerve-sparing procedure (yes/no), bladder-neck sparing procedure (yes/no), pelvic lymph node dissection (yes/no), and mean estimated blood loss (EBL). Postoperative variables included final Gleason score, multifocal cancer (yes/no), margin status, location of positive margins, lymph node invasion (yes/no), pathologic T stage, mean hospital stay, number of transfusions, and 30-day complication rate.

### 2.2. Description of the Procedure

**Figure 1:**
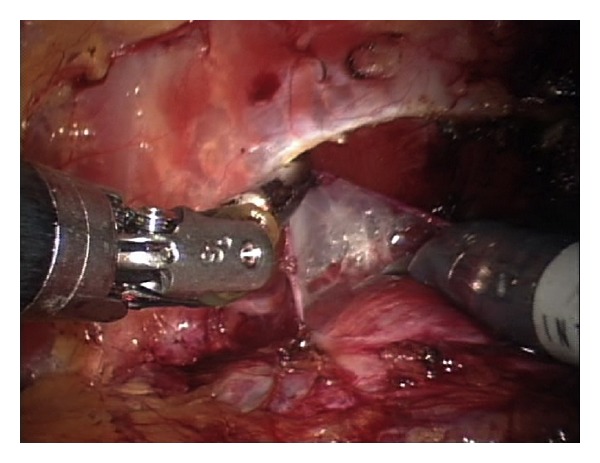
The lateral prostatic fascia is dissected sharply from the base of the prostate to its apex.

**Figure 2:**
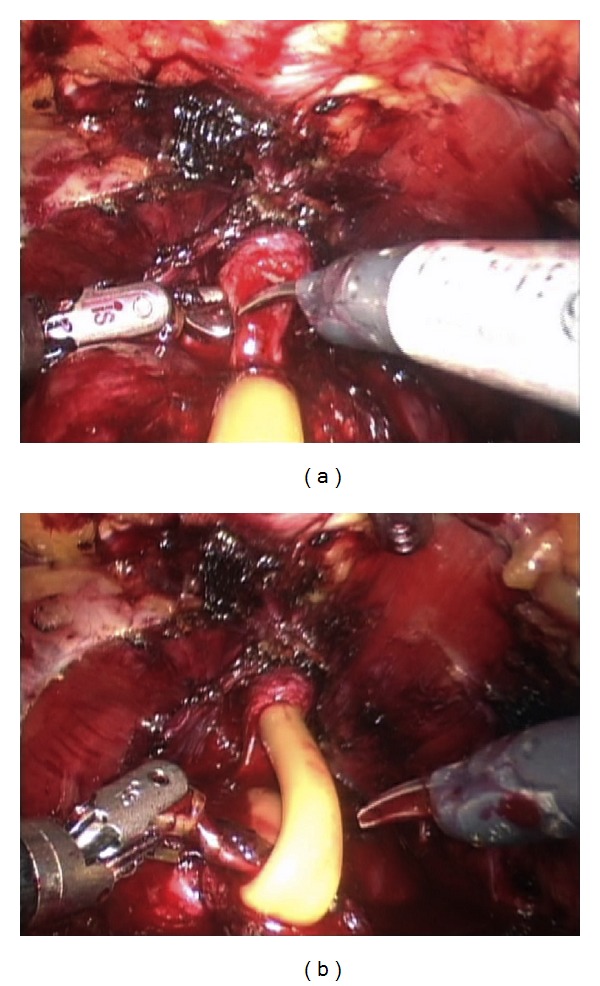
(a) With the NVB secured, the urethra is incised at the apex of the prostate, exposing the Foley catheter. (b) The posterior urethra is transected with subsequent retrograde dissection of the prostate off the rectum.

**Figure 3:**
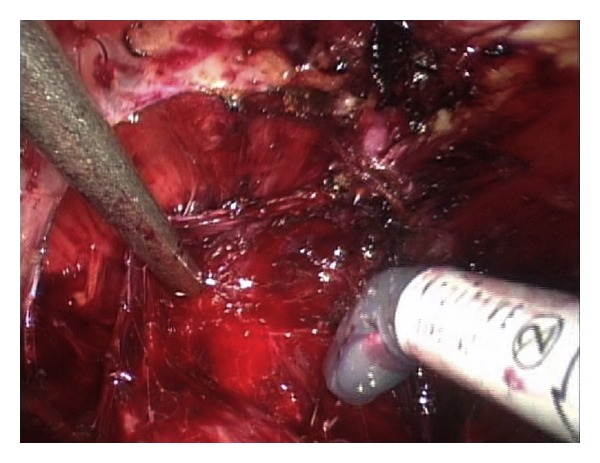
The prostate is dissected in a retrograde fashion until the seminal vesicles and vas deferens are reached.

We have previously presented the details of this technique [7] in a video submission. In summary, the bladder is dissected off the anterior abdominal wall and the endopelvic fascia is subsequently opened. The dorsal venous complex is controlled using a 0 Vicryl suture on a GS-21 needle in a figure-of-eight fashion. A back-bleeding 2-0 Vicryl stitch is then placed on the anterior surface of the prostate and the lateral prostatic fascia is dissected sharply from the base of the prostate to its apex (Figure 1). Using sharp dissection, the NVB is dissected off the apex of the prostate and with the NVB secured the urethra is incised at the apex of the prostate, exposing the Foley catheter (Figure 2(a)). The catheter is pulled inside the pelvis and three hem-o-lok clips are applied in order to prevent the balloon from deflating. The extracorporeal (proximal) end of the Foley catheter is then cut and is extended into the pelvis. The third arm of the robot is used to apply cephalad tension on the proximal end of the Foley catheter, providing exposure of the urethra posteriorly. The urethra is then incised posteriorly and the prostate is dissected off the rectum in a retrograde fashion using sharp dissection (Figure 2(b)). Subsequently, the prostatic pedicles are controlled using hem-o-lok clips or bipolar cautery and dissection is performed along the vesicoprostatic junction anteriorly until the Foley catheter is encountered. The Foley balloon is then drained by partially cutting the catheter and the distal end of the Foley catheter is brought out through the vesicoprostatic incision. Gentle caudal traction is applied to both ends of the Foley catheter using the third robotic arm, providing exposure of the vesicoprostatic junction posteriorly. Dissection along the vesicoprostatic junction is continued posteriorly, with exposure of the vas deferens and seminal vesicles (Figure 3). The vas deferens and seminal vesicles are isolated and after application of hem-o-lok clips the vas deferens is severed. Hem-o-lok clips are used to control what remains of the prostatic pedicles and an endocatch bag is used to extract the specimen. The vesicourethral anastomosis is subsequently performed using a running 3-0 barbed Maxon suture.

## 3. Results

All 18 patients had successful procedures without the need for conversion to open surgery or modification of the planned robotic technique. Pre-, intra-, and postoperative characteristics are described in Table 1. Complete or partial nerve sparing was performed in 16 of 18 patients (89%) (“partial” or “complete” nerve sparing was subjective and was based on the surgeon's intraoperative judgment). Intraoperatively, the mean estimated blood loss was less than 150 cc (range 40–300) and no patient required a blood transfusion. The mean total surgery time was 198 minutes (range 150–300) with a mean robotic operating console time of 168 minutes (range 118–265). Three of 18 patients (16.7%) had a positive surgical margin on final pathology. Two of these were found at the bladder neck and the third was found at the apex.

All patients had an uncomplicated postoperative course and were discharged home by a median of 30 hours postoperatively. Upon followup, two patients developed deep-vein thrombosis (Clavien class II complications), including one patient that had a symptomatic pulmonary embolus. Both patients were at high risk of DVT. The first patient had a factor V deficiency with history of spontaneous DVT and pulmonary embolus (on both enoxaparin and warfarin with an INR of 3.2 at the time of DVT). The second patient had a history of superficial thrombophlebitis and varicose veins and was on prophylactic enoxaparin. Both patients were anticoagulated and recovered from their complications without further incident. Upon a median followup of one year, 10 patients were continent using no pads, 5 patients were using only one pad per day, and 3 patients were lost for F/U. Potency defined as Sexual Health Inventory in Men (SHIM) score higher than 20 was achieved in 10 patients. Four patients had a SHIM score of 3, 8, 10, and 15, respectively, and we could not obtain the records on the rest. PSA was undetectable (PSA < 0.01) in all but one patient.

## 4. Comment

Radical retropubic prostatectomy with retrograde NVB preservation was first described by Walsh and Donker [8] decades ago and has become the gold standard for open treatment of localized prostate cancer. Most open RRPs are performed following the retrograde approach and thus most urologists are familiar and confident performing this procedure. Since the initial proposal of a laparoscopic approach to radical prostatectomy in 1992, most urologists have adapted antegrade dissection as the standard approach for preservation of the NVBs [9]. Rassweiler et al. [10] described a retrograde technique in which the periprostatic fascia was initially incised at the apex of the prostate and the NVBs dissected off, similar to our current proposal. Since that time, however, most surgeons have adopted the antegrade approach as described by Guillonneau and Vallancien [5] by making a lateral incision in the periprostatic fascia after division of the prostatic pedicles and dissection of the NVBs was carried out from these lateral incisions in an antegrade fashion.

Robotic surgery evolved as a refinement of the laparoscopic approach [1] and, in most cases, imitation of the laparoscopic technique was subsequently adopted [11, 12]. However, robotic instrumentation for laparoscopic prostatectomy introduces several contemporary advances to accelerate learning for novice laparoscopic surgeons, notably 3-dimensional vision and more natural surgical manipulation [13]. This is mostly due to articulated instruments with six degrees of freedom that allow dissection in different angles in a manner similar to the open approach. We hypothesize that, for surgeons who are familiar with the open retrograde approach, our technique allows for more seamless adoption of robotic surgery because of its close similarity to the open approach. We believe this to be especially true for the skilled open but laparoscopically inexperienced surgeon. Furthermore, by early dissection, isolation, and thus protection of the NVBs at the apex of the prostate (where it is most commonly injured), one might expect a better preservation of erectile function. This is especially true considering how closely and complexly related the NVBs are to the base of the prostate. Thus, there exists a chance of inadvertent trauma to the NVBs during an antegrade approach to nerve preservation. Without an initial incision in the endopelvic fascia, the precise course of the NVBs is not well visualized during antegrade dissection. Moreover, the fascial layers that surround the prostate can be precisely identified using the retrograde approach. Due to this superior visualization, all of the important key steps to performing open nerve-sparing radical prostatectomy can be accomplished, including high incision in the endopelvic fascia and hemostatic control of the vascular branches between the NVBs and the prostate.

The benefits of the retrograde approach to nerve sparing have been previously compared to the antegrade approach for robotic prostatectomy. Ko et al. [14] reported on 501 potent men (SHIM score > 21) who underwent bilateral full nerve sparing who were followed up for at least one year. After propensity score matching, 172 patients who underwent antegrade nerve sparing were compared to 172 patients who underwent retrograde nerve sparing. They found no difference in positive margin rate; however, the potency rate at 3, 6, and 9 months was significantly higher for retrograde nerve sparing (65% versus 81%; 72% versus 90%; 85% versus 93%, resp.). In a multivariable model, the approach to nerve sparing (retrograde versus antegrade) was an independent predictor of potency.

As with any other surgical procedure, patient selection is key and we believe this to be especially true in one's early experience. We selectively offered this technique to patients with smaller prostates (less than 50 cc) who had low volume and low grade cancer with higher SHIM scores (>17). We also initially utilized the 30-degree lens in order to optimize exposure and visualization of the posterior surface of the prostate. In the evolution of this technique, we now use the 0-degree lens for the entire procedure and feel that this does not compromise visibility. We found that our main technical limitation was the initial retrograde dissection of the apical portion of the prostate off the rectum. We were initially apprehensive about the possibility of rectal injury as a result of decreased proprioception experienced during robotic surgery, as well as iatrogenic positive apical margins. As such, we have recently modified our technique over the last five operations to begin with the posterior dissection, starting at the level of the vas deferens and seminal vesicles. That enabled us to separate the posterior surface of the prostate off the rectum before starting the apical division. Similar to the Montsouris technique [5], the procedure begins by incising the peritoneum in the midline at the level of the second inferior peritoneal arch to identify the vas deferens. The vasa are incised and the seminal vesicles are secured and mobilized. Next, Denonvilliers' fascia is opened and the dissection plane is continued posterior to the prostate and anterior to the rectum until the rectourethralis muscle is encountered. We then continue with the anterior approach and dissect the bladder attachments from the anterior abdominal wall.

## 5. Conclusions

Robotic retrograde radical prostatectomy is a novel robotic approach that attempts to replicate the familiar open technique. Our early experience with retrograde robotic radical prostatectomy demonstrates the feasibility of this approach with early outcomes comparable to the contemporary laparoscopic antegrade approach. Long-term study with a greater number of patients will be necessary to fully evaluate the oncologic and functional outcomes using this technique.

## Figures and Tables

**Table 1:** Preoperative, operative, and postoperative patient demographics and outcomes for 18 patients undergoing retrograde robotic-assisted laparoscopic prostatectomy.

Patient and procedure variables	Outcome
Preoperative	
Mean age, years (range)	59 (42–76)
Mean BMI, kg/m^2^ (range)	28 (21–37)
Mean PSA, ng/mL (range)	6.0 (1.2–23.0)
Mean IPSS (median)	8 (8)
Mean SHIM (median)	19 (23)
Biopsy Gleason score	6 (*N* = 9)
	7 (*N* = 7)
	8 (*N* = 2)
Clinical T stage	T1c (*N* = 14)
	T2a (*N* = 4)
Operative	
Mean operative time, min (range)	198 (150–300)
Mean robotic console time, min (range)	168 (118–265)
Nerve-sparing procedure	Total (*N* = 10)
	Partial (*N* = 6)
	None (*N* = 2)
Bladder-neck sparing procedure	Yes (*N* = 16)
	No (*N* = 2)
Pelvic lymph node dissection	Yes (*N* = 3)
	No (*N* = 15)
Mean EBL, mL (range)	142 (40–300)
Postoperative	
Final Gleason score	6 (*N* = 8)
	7 (*N* = 9)
	8 (*N* = 1)
Multifocal cancer	Yes (*N* = 14)
	No (*N* = 4)
Margin status	Negative (*N* = 15)
	Positive (*N* = 3)
Location of positive margin	Bladder neck (*N* = 2)
	Apex (*N* = 1)
Lymph node invasion	Yes (*N* = 1)
	No (*N* = 2)
	Unknown (*N* = 15)
Pathologic T stage	T2a (*N* = 4)
	T2c (*N* = 11)
	T3a (*N* = 1)
	T3b (*N* = 2)
Mean hospital stay, days (range)	2 (1–3)
Transfusions	None
30-day complications	*N* = 2*

BMI: Body Mass Index, PSA: Prostate Specific Antigen, IPSS: International Prostate Symptom Score, SHIM: Sexual Health Inventory for Men, and EBL: estimated blood loss.

*Both patients with deep-vein thrombosis.
